# A Graphene Oxide-Based Fluorescent Method for the Detection of Human Chorionic Gonadotropin

**DOI:** 10.3390/s16101699

**Published:** 2016-10-13

**Authors:** Ning Xia, Xin Wang, Lin Liu

**Affiliations:** 1 College of Chemistry and Chemical Engineering, Anyang Normal University, Anyang 455000, China; xianing82414@csu.edu.cn; 2 Henan Province of Key Laboratory of New Optoelectronic Functional Materials, College of Chemistry and Chemical Engineering, Anyang Normal University, Anyang 455000, China; wangx933@nenu.edu.cn

**Keywords:** graphene oxide, fluorescent biosensors, peptide aptamer, human chorionic gonadotropin, antibody-free

## Abstract

Human chorionic gonadotropin (hCG) has been regarded as a biomarker for the diagnosis of pregnancy and some cancers. Because the currently used methods (e.g., disposable Point of Care Testing (POCT) device) for hCG detection require the use of many less stable antibodies, simple and cost-effective methods for the sensitive and selective detection of hCG have always been desired. In this work, we have developed a graphene oxide (GO)-based fluorescent platform for the detection of hCG using a fluorescein isothiocyanate (FITC)-labeled hCG-specific binding peptide aptamer (denoted as FITC-PPLRINRHILTR) as the probe, which can be manufactured cheaply and consistently. Specifically, FITC-PPLRINRHILTR adsorbed onto the surface of GO via electrostatic interaction showed a poor fluorescence signal. The specific binding of hCG to FITC-PPLRINRHILTR resulted in the release of the peptide from the GO surface. As a result, an enhanced fluorescence signal was observed. The fluorescence intensity was directly proportional to the hCG concentration in the range of 0.05–20 IU/mL. The detection limit was found to be 20 mIU/mL. The amenability of the strategy to hCG analysis in biological fluids was demonstrated by assaying hCG in the urine samples.

## 1. Introduction

Human chorionic gonadotropin (hCG) is a glycoprotein hormone produced by the embryo and presented in the blood and urine of pregnant women [1]. Recently, elevated levels of hCG were found in many cancerous tumors, such as prostate cancer, testicular cancer, trophoblastic cancer and gestational choriocarcinoma [2]. Thus, hCG can be regarded as a biomarker for the diagnosis of pregnancy and some cancers. Because the lateral-flow immunoassay (the most commonly used method for hCG detection) has trouble accurately quantifying the level of hCG, a few new techniques have been made recently to determine hCG in blood and urine, such as enzymelinked immunosorbent assay (ELISA) [3], fluorescent immunoassay [4], immunochromatography [5], photoluminescence [6,7], surface plasmon resonance (SPR) [8] and electrochemical immunosensors [9,10,11,12,13,14,15,16,17,18,19,20]. These methods are sensitive and selective, but they are usually expensive, time-consuming and labor intensive and require the use of less stable antibodies. Moreover, the drive to produce disposable Point of Care Testing (POCT) devices uses a lot of antibodies, much more than in test kits used in a medical laboratory. This is by virtue of the very nature of design, sample handling, and equipment used by the skilled laboratory technician, which is not available to the laboratory unskilled user of POCT devices. However, there is a question in manufacturing terms of the consistence of biologically produced antibody batches and supply to meet the demand for POCT devices.

Of the alternatives to antibody-based sensing techniques, aptamer-based methods have become popular over the past decade. Recently, peptide aptamers have attracted great attention as promising candidates to replace antibodies since they are more stable and resistant to harsh environments and can be readily prepared with the desired sequences to bind the specific targets. Using the in vitro screening techniques, a large number of engineered peptide aptamers have been found and used as the recognition elements for biosensing [21,22,23,24,25]. Also, with the phage display technique, Yang’s group found an hCG-binding peptide aptamer (K_D_ = 0.9 nM) with a sequence of PPLRINRHILTR [2]. The findings gave the researchers a hint that the peptide could be used as an hCG-receptor for design of antibody-free biosensors. Typically, Lin and co-workers have developed two colorimetric biosensors based on the specific interaction between peptide aptamer and hCG and the good catalytic or optoelectronic properties of gold naoparticles (AuNPs) [26,27]. This AuNPs-based colorimetric sensing technique is simple and does not require modification of any analyte-binding molecules onto AuNPs. However, the unmodified AuNPs-based colorimetric assays show low sensitivity and poor anti-interference ability for protein assays in biological samples because the presence of some matrix components in biological fluids may protect or promote the aggregation of bare AuNPs [26,27].

**Scheme 1:**
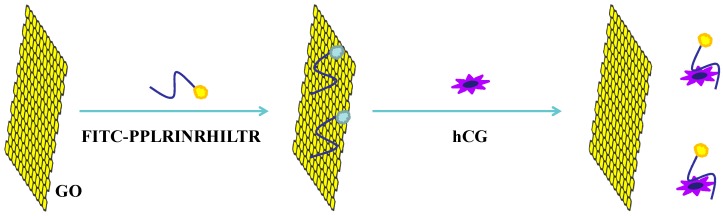
Schematic illustration of the GO-based fluorescent method for hCG detection with a peptide of FITC-PPLRINRHILTR as the probe.

It has been suggested that graphene oxide (GO) exhibits extraordinarily high quenching ability toward fluorescently labeled (e.g., dye, quantum dots or metal nanoclusters) DNA and peptides due to the prominent nanoscale–surface energy transfer effect from the fluorophore to GO [28,29,30,31,32,33]. Thus, many GO-based fluorescent chem/bio-sensors have been developed for monitoring the enzymatic activities [34,35,36,37,38,39], measuring the levels of various analytes including nucleic acids, proteins, metal ions and small molecules [40,41,42,43,44], and imaging of cells as well as animals [45,46]. Based on the high quenching ability of GO and the specific aptamer–target interaction, several groups have reported the detection of proteins (e.g., thrombin, cyclin A2, amyloid-β oligomers, α-bungarotoxin and antibodies) with the dye-labeled DNA or peptide probes as the recognition elements [47,48,49,50,51]. In a typical detection model, the fluorescence of a dye-labeled probe would be quenched when it was absorbed onto the surface of GO. However, the specific binding of a target protein to the fluorescently labeled probe would induce the release of the probe from the GO surface, thus resulting in the fluorescence recovery. In the present work, we found that the fluorescently labeled hCG-binding peptide can adsorb onto the surface of GO (Scheme 1). Consequently, the fluorescence of the peptide was quenched effectively through the energy-transfer or electron-transfer processes. However, with the addition of hCG, the specific binding of hCG to the peptide probe resulted in the release of the peptide from the GO surface, thus leading to the recovery of the fluorescence signal. Based on this fact, we have developed a GO-based fluorescent platform for the detection of hCG in the urine samples.

## 2. Materials and Methods

### 2.1. Reagents and Materials

**Figure 1:**
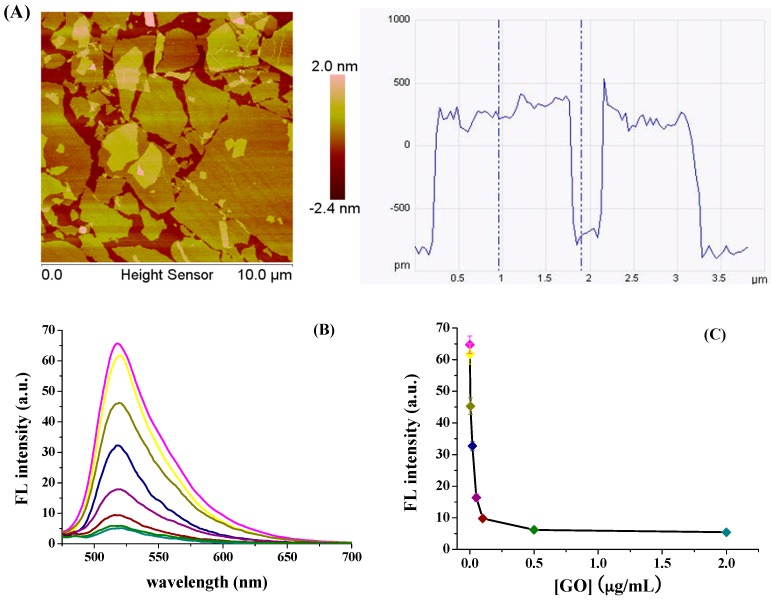
(**A**) AFM image and lateral size of GO; (**B**) Fluorescence spectra of 50 nM FITC-PPLRINRHILTR in the presence of different concentrations of GO; (**C**) Fluorescence intensity of FITC-PPLRINRHILTR versus the concentration of GO.

Bovine serum albumin (BSA), thrombin, immunoglobin G (IgG), hCG, KH_2_PO_4_, and K_2_HPO_4_ were purchased from Sigma-Aldrich (Shanghai, China). Recombinant human erythropoietin (rHuEPO) was provided by BioVision, Inc. (Milpitas, CA, USA). Beta-subunit of hCG (β-hCG) was obtained from YuduoBio Co., Ltd. (Shanghai, China). Single layer GO with oxygen content of 35%–40% was provided by Nanjing XFNANO Materials Tech Co., Ltd. (Nanjing, China). The Atomic Force Microscope (AFM) image shows that the thickness of GO is 0.8–1.2 nm and the lateral size is 0.5–5 μm (Figure 1A). The peptide probe labeled with fluorescein isothiocyanate (FITC) (a well-known fluorescent reporter for design of GO-based fluorescent chem/bio-sensors) in the N-terminal (FITC-PPLRINRHILTR) was obtained from Synpeptide Co., Ltd. (Shanghai, China). The serum sample containing 16.14 mIU/mL follicle-stimulating hormone (FSH), 11.82 mIU/mL luteotropic hormone (LH) and 3.41 mIU/mL thyroid stimulating hormone (TSH) from one woman donor (35 years old) and the urine samples from one woman donor (33 years old) were provided by Anyang Maternal and Child Heath Care Hospital (Anyang, China) and were stored at −18 °C for use. Amino acids used in this work were provided by Sangon Biotech. Co., Ltd. (Shanghai, China). The peptide at the concentration of 1 mM was dissolved with deionized-water and diluted to the desired concentration with a phosphate-buffered saline solution (PBS buffer, 20 mM, pH 7.2).

### 2.2. Quenching Studies

To determine the quenching efficiency of GO to FITC-PPLRINRHILTR, 50 μL of GO at a given concentration was mixed with 100 μL of FITC-PPLRINRHILTR. Then, 50 μL of PBS was added to the mixed solution for fluorescence measurement. Fluorescence spectra was collected on a Varian Cary fluorescence spectrometer with an excitation wavelength of 470 nm. The emission wavelength was taken with a slit of 5 nm. To determine the influence of amino acid and protein on the quenching efficiency of GO to FITC-PPLRINRHILTR, 50 μL of amino acid or protein was added into 100 μL of FITC-PPLRINRHILTR, followed by the addition of 50 μL of GO for fluorescence measurement.

### 2.3. Detection of hCG

For the assay of hCG, FITC-PPLRINRHILTR was first mixed with GO for 10 min to form the FITC-PPLRINRHILTR/GO complex. Then, 50 μL of hCG at a given concentration was added to 150 μL of the prepared FITC-PPLRINRHILTR/GO solution. After incubation at room temperature for 15 min, the fluorescence of the mixed solution was collected as aforementioned method. For the assays of serum or hCG in urine, the samples were centrifuged at 1300 rpm for 5 min. Then, 50 μL of the supernatant were taken out and added to 50 μL of PBS buffer, followed by the addition of 100 μL of the prepared FITC-PPLRINRHILTR/GO solution. Other procedures for the determination of hCG in urine samples were the same as those for the assay of hCG in the blank PBS.

## 3. Results and Discussion

### 3.1. Quenching Efficiency of GO to FITC-PPLRINRHILTR

We first investigated the interaction between FITC-PPLRINRHILTR and GO by monitoring the fluorescence change of FITC-PPLRINRHILTR in the presence of various concentrations of GO. It can be seen that the fluorescence intensity of FITC-PPLRINRHILTR decreased with the increase of GO concentration and reached the minimum value beyond 0.5 μg/mL (Figure 1B,C). The result indicated that the fluorescence of FITC-PPLRINRHILTR was quenched efficiently by GO. It has been demonstrated that the adsorption of peptide onto the GO surface depends upon the electrostatic and π–π interactions between the negatively charged GO and the positively charged amino acid residues (Lys, His, and Arg) as well as the aromatic-ring-containing hydrophobic amino acids (Trp, Tyr, and Phe) [36,49,52]. Note that there are three Arg and one His amino-acid residues within the peptide probe (FITC-PPLRINRHILTR). Thus, the adsorption of the peptide onto the GO surface should be attributed to the electrostatic interaction. The quenching efficiency of 2 μg/mL GO to FITC-PPLRINRHILTR was found to be 89.2% ± 3.6% by the formula (1 = F/F_0_) × 100%, where F and F_0_ represent the fluorescence intensity at 518 nm with and without the addition of GO.

**Figure 2:**
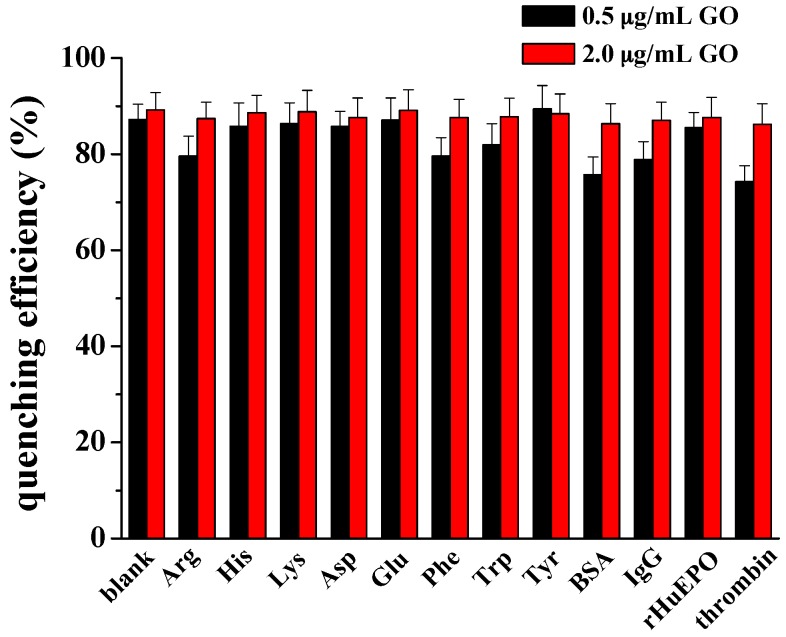
Effects of various amino acids and proteins on the quenching efficiency of 0.5 and 2 μg/mL GO to FITC-PPLRINRHILTR. The final concentrations of FITC-PPLRINRHILTR, amino acids and proteins were 50 nM, 50 μM and 10 ng/mL, respectively.

Some amino acids and proteins may also show the electrostatic and π–π interactions with GO. This maybe has a negative effect on the interaction of FITC-PPLRINRHILTR and GO. For this view, we studied the impact of hydrophilic or aromatic amino acids (Arg, His, Lys, Asp, Glu, Phe, Trp and Tyr) and proteins (BSA, IgG, rHuEPO and thrombin) on the quenching efficiency of GO to FITC-PPLRINRHILTR (Figure 2). Consequently, we found that Lys, His, Glu and Asp showed negligible effects on the quenching efficiency. However, the presence of Arg, Phe, Trp, Tyr and the four tested proteins weakened the interaction of GO to FITC-PPLRINRHILTR, thus decreasing the quenching efficiency in different degree. The use of excess concentrations of GO may reduce the interference of some matrix components existing in biological samples. Herein, we found that the fluorescence of FITC-PPLRINRHILTR could be quenched effectively by a high concentration of GO even in the presence of these tested interferences. Thus, in the following detection assays, GO at an excess concentration of 2 μg/mL was used for the detection assay.

### 3.2. hCG Detection

**Figure 3:**
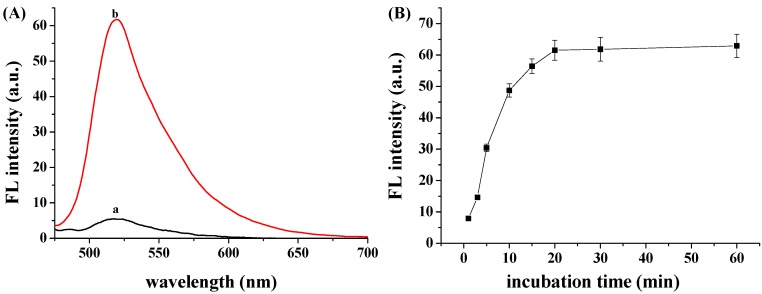
(**A**) Fluorescence spectra of FITC-PPLRINRHILTR/GO in the absence (curve a) and presence (curve b) of hCG. The concentrations of FITC-PPLRINRHILTR and hCG were 50 nM and 50 IU/mL, respectively; (**B**) Fluorescence restoration of FITC-PPLRINRHILTR/GO by hCG as a function of time.

The fluorescence spectra of FITC-PPLRINRHILTR/GO in the absence (curve a) and presence (curve b) of hCG are shown in Figure 3A. It can be observed that the fluorescence signal increased in the presence of hCG. The result demonstrated that the specific binding of hCG to the peptide resulted in the desorption of FITC-PPLRINRHILTR from the GO surface. For three parallel experiments, the fluorescence intensities were found to be 59.2, 61.8 and 62.1, suggesting a good reproducibility. The result indicated that the FITC-PPLRINRHILTR/GO complex is a good probe for the detection of hCG. We also investigated the effect of the incubation time of hCG with the probe on the fluorescence intensity. As shown in Figure 3B, the fluorescence intensity increased gradually within 20 min incubation. The result indicated that the competitive binding of hCG with GO for FITC-PPLRINRHILTR diminished the GO and peptide contact, leading to the gradual desorption of peptide from the GO surface.

### 3.3. Sensitivity to hCG

**Figure 4:**
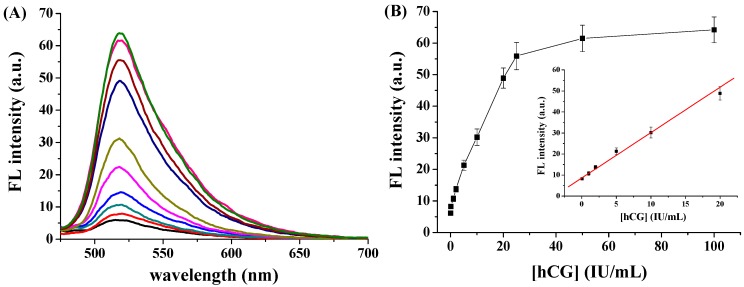
(**A**) Fluorescence spectra of FITC-PPLRINRHILTR/GO in the presence of different concentrations of hCG; (**B**) Dependence of fluorescence intensity on hCG concentration. The inset shows the linear segment in the range of 0.05 to 20 IU/mL.

We determined the sensitivity and detection limit of this method. Figure 4A shows the fluorescence spectra of FITC-PPLRINRHILTR/GO in the presence of different concentrations of hCG. The fluorescence intensity increased with increasing hCG concentration. As shown in Figure 4B, the fluorescence intensity was linearly proportional to the hCG concentration in the range of 0.05–20 IU/mL. The linear regression equation is F = 8.6 + 2.2 [hCG] (IU/mL) (R = 0.993). The relative standard deviations (RSDs, shown as the error bars in Figure 4B) for assays of the different concentrations of hCG samples are all less than 8.5%, further indicating a good reproducibility of this method. The analytical performances of this method were compared to those achieved by other methods (Table 1). The detection limit of 20 mIU/mL was comparable to (or even lower than) that achieved by immunochromatography, photoluminescence, surface plasmon resonance, fluorescent immunoassays, liquid crystal-based assay and AuNPs-based colorimetric assays. Although the detection limit is higher than that achieved by ELISA and some electrochemical methods, our method obviates the pre-modification of nanomaterials, requires very simple sample handling, and does not need to use the relatively expensive and less stable antibodies.

### 3.4. Selectivity and Real Sample Assay

**Figure 5:**
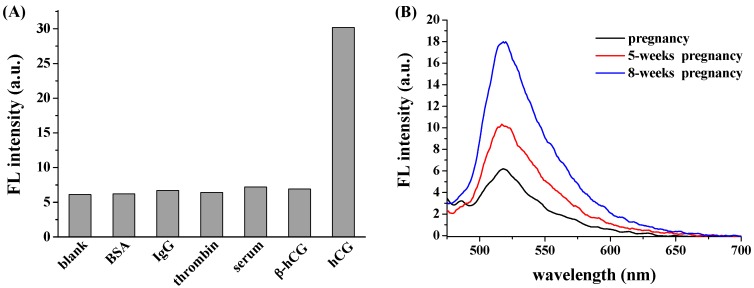
(**A**) Selectivity of the proposed sensing strategy. The final concentrations of BSA, IgG, thrombin, β-hCG and hCG were 10 ng/mL, 10 ng/mL, 10 ng/mL, 25 ng/mL and 10 IU/mL, respectively; (**B**) Fluorescence spectra of FITC-PPLRINRHILTR/GO for the detection of hCG in urine samples.

Biological fluids may contain many proteins (e.g., antibodies, proteases, glycoproteins and other common proteins). To explore the selectivity of the proposed method, three interfering proteins (BSA, IgG, thrombin) and serum sample containing FSH, LH and TSH were tested. Moreover, beta-subunit of hCG (β-hCG) is also abundant in pregnancy sample. Thus, the specificity of the aptamer to β-hCG was also investigated. As shown in Figure 5A, compared to the control, none of these interferences caused a significant increase in the fluorescence intensity, demonstrating that the established fluorescent platform showed extraordinary selectivity towards hCG. The high selectivity could be principally attributed to the strong and specific interaction between hCG and its binding peptide (K_D_ = 0.9 nM) [2].

The good selectivity and sensitivity of the method encouraged us to quantify hCG in the biological samples. Figure 5B shows the fluorescence spectra of FITC-PPLRINRHILTR/GO in the presence of urine samples provided by one female donor pregnant with different periods. It can be observed that the fluorescence signal is close to the background level for assay of the urine from the donor without pregnancy, demonstrating that no detectable hCG was found in the urine. Furthermore, we found that the recoveries for the added hCG with 1, 2 and 20 IU/mL were 97.4% ± 7.6%, 106.3% ± 8.1% and 96.6% ± 6.3%, respectively. The result also indicated that the matrix components in blank urine showed no or poor interaction with the aptamer. More interestingly, the fluorescence signal increased greatly at the case of 5 weeks and 8 weeks pregnancy. Based on the linear curve presented in Figure 4B, the concentrations of hCG in the two urine samples were calculated to be 2.54 ± 0.27 IU/mL and 16.7 ± 1.46 IU/mL, respectively. The result indicated that elevated level of hCG was presented in the urine with long-time pregnancy. Thus, the proposed GO-based biosensor may offer an alternative means for hCG detection in clinical investigations.

## 4. Conclusions 

In conclusion, we presented an antibody-free GO-based fluorescent platform for the detection of hCG using a fluorescently labeled hCG-specific binding peptide as the probe, which can be manufactured cheaply and consistently. Compared with the previously reported optical and electronic immunoassays, our method requires simple operating procedure and obviates the use of less stable antibodies. The detection limit is comparable to that achieved by the AuNPs-based colorimetric assays with the hCG-specific binding peptide as the probe. However, our method shows good anti-interference ability for assays of biological samples, thus facilitating the detection of hCG in urine samples. Furthermore, this was coupled in a process format that directly links binding of hCG to aptamer to a colourimetric quenching competitive quantitative signal. This can form the basis of an inexpensive, quantitative hCG disposable POCT type device. This same format can also be used when the aptmer is replaced to quantitatively measure other important biomolecules such as carcino-embryonic antigen (CEA), cancer antigen 125 (CA-125) and alpha-fetoprotein (AFP) all in a POCT device.

## Figures and Tables

**Table 1:** Analytical performances of various methods for hCG detection.

Materials	Methods	Detection Limit	Linear Range	Reference
SPAAB-HRP/anti-hCG	ELISA	0.012 mIU/mL	-	[3]
anti-hCG/ZnO	PL	2 ng/mL	2–20 ng/mL	[6]
anti-hCG/CdSe-ZnS QDs	PL	0.5 mIU/mL	-	[7]
anti-hCG/AuNPs	IGCA	5 ng/mL	10–600 ng/mL	[5]
anti-hCG/gold film	SPR	<500 ng/mL	-	[8]
peptide aptamer	LC	1 IU/mL	12.5–100 mIU/mL	[2]
PF@SiO_2_-Ab2 and Fe_3_O_4_@PANI-Ab1	fluorescence	3 pg/mL	0.01–100 ng/mL	[4]
peptide aptamer/AuNPs/4-nitrophenol	colorimetry	15 mIU/mL	15–750 mIU/mL	[26]
peptide aptamer/AuNPs	colorimetry	25 mIU/mL	25–1000 mIU/mL	[27]
anti-hCG/Au-MWCNTs/GS/GCE	DPV	0.0026 mIU/mL	0.005–500 mIU/mL	[13]
anti-hCG/AuE	SWSV	15 pM	15–300 pM	[10]
HRP-Ab2/hCG/Ab1/nafion/GCE	CA	11.2 mIU/mL	200 mIU/mL	[9]
anti-hCG/CS/graphene-SPE	EIS	0.016 ng/mL	0.1–25 ng/mL	[19]
anti-HCG/ FPD/GCE	EIS	0.03 ng/mL	0.1–10 ng/mL	[14]
anti-hCG/Pd@SBA-15/TH/HSO_3_-GS/GCE	CV	8.60 pg/mL	0.01–16.00 ng/mL	[20]
GCE/GS/NPG/anti-hCG	CV	0.034 ng/mL	0.5–40.00 ng/mL	[11]
hCG/HRP-anti-hCG/sol–gel/GE	DPV	0.3 mIU/mL	0.5–50 mIU/mL	[17]
Peptide aptamer/GO	fluorescence	20 mIU/mL	0.05–20 IU/mL	This work

SPAAB-HRP, horseradish peroxidase (HRP)-loaded nanospherical poly(acrylic acid) brushe (SPAAB); anti-hCG, human chorionic gonadotropin antibody; QDs, quantum dots; PF@SiO_2_, poly[(9,9-bis(3′-((N,Ndimethylamino)N-ethylammonium)propyl)-2,7-fluorene)-alt-2,7-(9,9-p-divinyl-benzene)-alt-fluorene)-alt-2,5-dimethyl-p-phenylenediamine] coated SiO_2_ nanoparticles; Fe_3_O_4_@PANI, polyaniline coated Fe_3_O_4_ nanoparticles; MWCNTs, multiwalled carbon nanotubes; CS, graphene sheets; GCE, glassy carbon electrode; AuE, gold electrode; Ab, antibody; SPE, screen printed electrode; FPD, 2-(4-Formylphenyl)[60]fulleropyrrolidine; HSO_3_-GS, Functionalized graphene nanomaterial with introduced -SO_3_ groups; SBA-15, one of mesoporous silicas with uniform tubular channels; TH, thionine; NPG, nanoporous gold; PL, photoluminescence; IGCA, immunogold chromatographic assay; LC, liquid crystal assay; DPV, differential pulse voltammetry; SWSV, square-wave stripping voltammetry; CA, chronoamperometry; EIS, electrochemical impedance spectroscopy.
